# Neurological Disorders Associated with Glutamic Acid Decarboxylase Antibodies

**DOI:** 10.7759/cureus.4738

**Published:** 2019-05-23

**Authors:** Kimberly Herard, Javed L Khanni, Kettia Alusma- Hibbert, Courtland R Samuels, Patricio S Espinosa

**Affiliations:** 1 Internal Medicine, Florida Atlantic University Charles E. Schmidt College of Medicine, Boca Raton, USA; 2 Neurology, Florida Atlantic University Charles E. Schmidt College of Medicine, Boca Raton, USA; 3 Neurology, Marcus Neuroscience Institute at Boca Raton Regional Hospital, Boca Raton, USA; 4 Emergency Medicine, Florida Atlantic University Charles E. Schmidt College of Medicine, Boca Raton, USA

**Keywords:** anti gad antibodies, neurological symptoms, myasthenia gravis, guillan barre syndrome, paraneoplastic

## Abstract

Anti-glutamic acid decarboxylase (GAD) antibodies have been discovered in a variety of neurological syndromes with unique presentations. These syndromes include limbic encephalitis (LE), stiff person syndrome (SPS), opsoclonus-myoclonus-ataxia syndrome, cerebellar ataxia, status epilepticus, and palatal myoclonus among others. We present two patients who presented with Guillain-Barré (GBS) and myasthenia gravis (MG) like syndromes, who were found to have anti-GAD antibodies. These case reports highlight the complex presentation of patients with neurological disorders associated with anti-GAD antibodies. The proper identification of anti-GAD antibody’s presence has proven to be beneficial in treatment and provide enhanced quality of life.

## Introduction

Glutamic acid decarboxylase (GAD) enzymes are found in the central nervous system (CNS). GAD can exist in two different isoforms, GAD65 and GAD67. GAD enzymes are essential for the formation of gamma aminobutyric acid (GABA), an inhibitory neurotransmitter in the brain [1]. Antibodies against the GAD enzyme result in lack of GABA, therefore, patients with these antibodies can exhibit motor and cognitive symptoms.

Anti-GAD antibodies have been associated with numerous neurological conditions that have a wide variety in presenting symptoms. These conditions include: limbic encephalitis, stiff person syndrome (SPS), opsoclonus-myoclonus-ataxia syndrome, cerebellar ataxia, and status epilepticus among others [2-9]. Patients with limbic encephalitis often present with cognitive and memory impairments, psychiatric symptoms, and seizures [2]. SPS can present with an axial distribution of stiffness and painful muscle spasms in the paraspinal muscles [3]. Patients with opsoclonus-myoclonus-ataxia syndrome present with involuntary eye movements, ataxia, myoclonus, and dystonia [4]. Whereas patients with cerebellar ataxia present with dysarthria, gait and balance disorders, and limb ataxia [5]. Additionally, patients with refractory epilepsy, and no identifiable brain abnormalities, have been reported to have serum antibodies against neuronal antigens including GAD [6].

The current literature reports a wide array of neurologic conditions associated with anti-GAD antibodies. This should prompt us to include an anti-GAD antibody etiology in our differential when evaluating patients who present with complex neurologic and/or psychiatric symptoms. Furthermore, correct identification of anti-GAD antibodies in these conditions has proven to be beneficial in therapy. Here we add to the current body of literature by discussing the clinical presentation of two patients, who tested positive for anti-GAD antibodies, after presenting with signs and symptoms of Guillain-Barré syndrome (case 1) and myasthenia gravis (case 2).

## Case presentation

Case 1

A 61-year-old Caucasian male with a past medical history significant for attention-deficit/hyperactivity disorder, anxiety, pulmonary embolism, hypertension, protein C deficiency, Hashimoto’s thyroiditis and prior syncopal episodes presented to the emergency department (ED) with a chief complaint of multiple falls. He reported falling multiple times a day for four consecutive days prior to ED admission. He attributed his falls to new onset, bilateral, lower-extremity weakness which started four days prior and progressively worsened. He described his legs as feeling like “jello” when standing for long periods of time. The patient also noted associated symptoms of intermittent slurred speech, dizziness, and vertigo. Of note, family history was significant for amyotrophic lateral sclerosis (ALS).

Neurological exam revealed hyporeflexia in lower extremities bilaterally, slowing of finger-to-nose coordination on the left side, and ataxic gait with sustained posture. Remainder of the physical exam was within normal limits including 4/5 strength in all extremities, intact sensation and proprioception, and negative Romberg. Brain and spine imaging was normal.

The cerebrospinal fluid (CSF) analysis of a lumbar puncture showed two white blood cells (WBC), normal protein level and normal glucose level. Based on the initial presentation, and diagnostic results, the patient was presumptively diagnosed with Guillain-Barré syndrome (GBS) and started on intravenous immunoglobulin (IVIG) therapy. The patient had near resolution of symptoms after the first dose of IVIG. However, since the patient felt back to normal, he insisted on being discharged and did not complete the full course of IVIG. The patient was then lost to follow up.

**Figure 1:**
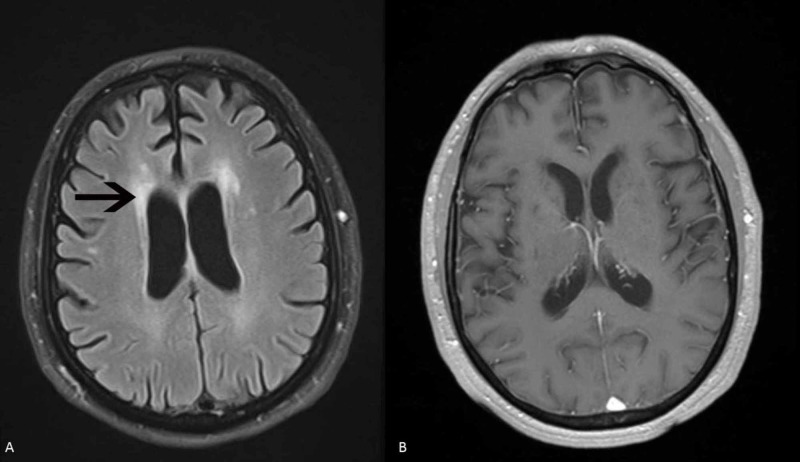
MRI of brain patient 1 (A) MRI of the brain FLAIR sequence shows mild ischemic gliotic changes bilaterally but no acute abnormalities. (B) T1 weighted image shows no enhancing lesions.

Two months later, the patient returned to the ED presenting with symptoms of weakness and recurrent falls. On this encounter, he reported the symptoms to be worse at night with increased weakness in upper extremities. Neurological exam was significant for decreased lower extremity reflexes and absence of patellar reflexes. At this point, clinical suspicions were raised for multifocal motor neuropathy, myasthenia gravis, Lambert-Eaton syndrome, and chronic inflammatory demyelinating polyradiculopathy syndrome (CIDP). Electromyography (EMG) revealed prolonged F-waves and denervation potentials in the lower extremities which is consistent with the clinical presentation of GBS. However, CSF studies were within normal limits. Further workup included a chest, abdominal, and pelvic computed tomography (CT) which demonstrated no abnormal findings. Brain magnetic resonance imaging (MRI) was within normal limits (Figure 1).

A paraneoplastic panel, however, revealed positive GAD65 antibodies with titers at 0.09 mmol/L (immunoprecipitation assay, Mayo Clinic, RR < 0.02 nmol/L). Also positive were striational antibodies at 1:480 (immunoprecipitation assay, Mayo Clinic, RR < 1:120 nmol/L), elevated thyroid stimulating hormone (TSH) at 11,900 mclU/mL), high thyroid peroxidase antibodies at 78.6 IntUnit/mL, thyroglobulin antibody at 117.5 IntUnit/mL, and elevated creatine kinase at 194 IntUnit/mL. Following the extensive workup, the patient received a full course of IVIG, 0.4 g/kg IV for five days, along with Solumedrol 250 mg intravenous twice a day for three days. The patient exhibited good treatment response. His symptoms have been well controlled with IVIG every three weeks.

Case 2

A 63-year-old Caucasian male with past medical history significant for refractory myasthenia gravis (MG), epilepsy and essential tremor, presented with chief complaints of various neurological issues. For the past 14 years, the patient had complaints of weakness, difficulty breathing, eye weakness, foot drop, weight loss associated with difficulty swallowing, and tremors. His treatment regimen at this time included plasmapheresis every other day initiated 14 years ago, two days of IVIG every three or four weeks, prednisone 10 mg daily, and tacrolimus 0.5 mg daily. He also had a trial with Mestinon but it was discontinued due to lack of improvement.

The patient presented to our clinic to establish care with the aim of continuing his current treatment regimen. On initial inpatient encounter, the patient still complained of generalized weakness, dyspnea, dysphagia and difficulty opening his eyes. He admitted to discontinuation of plasmapheresis exchange treatments two months prior due to experiencing weakness and fatigue following each treatment. On physical exam, he had ankle foot orthoses (AFO) in place and decreased muscle mass in bilateral lower extremities. Patient’s gait was slow and unsteady with use of cane for ambulatory support. No other neurological deficits were noted.

**Figure 2:**
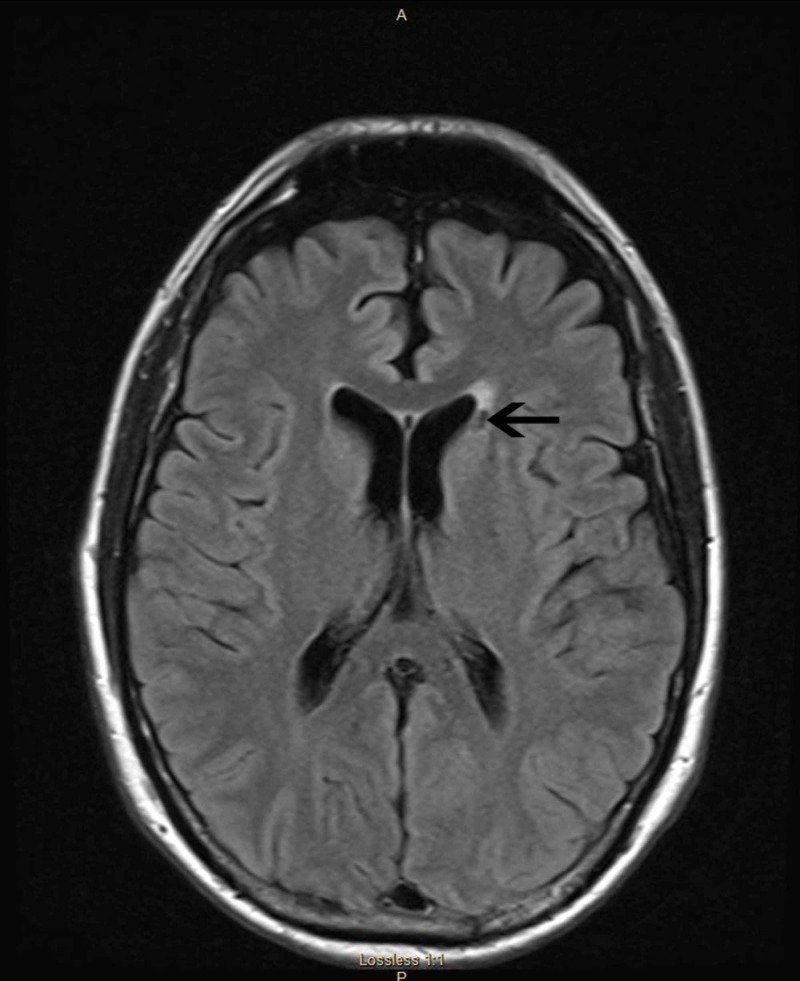
MRI of brain patient 2 MRI of the brain FLAIR sequence shows no acute abnormalities but evidence of a lacunar stroke in the left, just above the head of the caudate nuclei.

On further testing, MRI of brain, cervical spine, and lumbar spine imaging did not reveal abnormal findings (Figure 2).

Electroencephalogram (EEG) revealed focal left temporal sharp waves consistent with epilepsy diagnosis. Computed tomography (CT) of chest, abdomen, and pelvis revealed normal findings. Immunological assays were negative for binding, blocking, and modulating acetylcholine receptor antibodies (AChR-Ab). Serological testing was also negative for muscle-specific tyrosine kinase autoantibodies (MuSK). He tested positive for antithyroid antibodies concluding a new diagnosis of Hashimoto’s thyroiditis. Paraneoplastic panel did reveal patient’s serum was positive for anti-GAD antibodies at 63.2 mmol/L (immunoprecipitation assay, ARUP Lab, RR < 0.0-5.0).

We concluded that this patient did not have MG and was therefore weaned off tacrolimus and prednisone and plasmapheresis was discontinued. He was treated with a three-week course of IVIG with stable symptoms.

## Discussion

There is a growing body of anti-GAD antibody-associated neurologic syndromes with very diverse clinical presentations (Table 1). Adding to these presentations are our two cases in which patients presented with neurologic symptoms, mimicking established neuro-immunologic diseases, who tested positive for anti-GAD antibodies.

**Table 1:** Reported neurological disorders associated with anti-GAD antibodies GAD: Glutamic acid decarboxylase. [3,5,7-14]

Neurological Disorders	Clinical Presentation
Stiff-Person syndrome	Rigidity of axial and proximal muscles, intermittent muscle spasms, lumbar lordosis
Cerebellar ataxia	Dysarthria, limb ataxia, gait and balance disorder
Autoimmune Limbic Encephalitis	Acute or subacute seizures, memory loss, irritability, hallucinations, pathogenesis with symptoms localized to temporal lobes
Opsoclonus-myoclonus-ataxia syndrome	Involuntary, irregular eye movements; ataxia, myoclonus
Anti-GAD65 Antibody Associated Epilepsy	Convulsive disorders often refractory to treatment
Palatal myoclonus	Contractions of tensor veli palatini with possible development of tinnitus or ear clicking; often associated with cerebellar ataxia or brainstem lesion
Necrotizing Myelopathy	Paraneoplastic neurologic syndrome: Systemic cancer associated with neurologic disorders affecting spinal cord and cerebral cortex—ascending sensory deficits, sphincter dysfunction, tetraplegia
Motor Neuron Syndromes	Asymmetric motor dysfunction, subacute, progressive lower motor neuron weakness

The patient in case 1 initially presented with a GBS-like syndrome. Upon further workup, CSF studies did not reveal evidence of the classical GBS finding termed albuminocytological dissociation in which there is an elevated CSF protein level with normal CSF white blood cell counts. This finding is present in 50-66% of patients within the first week of symptoms and 75% following the third week of symptoms [15]. Although EMG results were consistent with GBS, the lack of albuminocytological dissociation in this patient made the diagnosis of GBS less likely. Similarly, our patient in case 2 presented with a history of MG refractory to treatment, which included Mestinon early in the disease process. Most recent serological assays were negative for AChR-antibodies and MuSK antibodies further demonstrating an incorrect diagnosis of MG despite similarities in clinical presentation. In both cases, anti-GAD antibodies were detected in the serum and are believed to play a role in the etiology of these neurologic disorders with very distinct clinical presentations.

The clinical presentation of patients positive for anti-GAD antibodies can vary greatly making the diagnosis difficult prior to confirmation following a paraneoplastic panel. Antibody attack against GAD65 and GAD67 enzymes produces a variety of cognitive and motor symptoms. As previously discussed, varying clinical manifestations of anti-GAD antibodies include motor symptoms such as muscle rigidity and spasms, limb ataxia, balance disorders, and myoclonus. Cognitive deficits include memory loss, irritability, and hallucinations. Various clinical presentations of anti-GAD antibodies also present with sensory defects. Further research is required to understand why variable presentations of anti-GAD antibodies exist clinically as evidenced in the unique symptoms presented in the above cases. However, early detection of anti-GAD antibodies is important in diagnosis and treatment of the various clinical symptoms seen in patients. As evident in case 1 above, anti-GAD antibody symptoms of ataxia, weakness, and slurred speech persisted for years until eventual diagnosis and treatment with IVIG led to relief of symptoms. Appropriate diagnosis of anti-GAD antibodies in case 2 occurred after a period of time on Mestinon for suspected MG, further demonstrating the illusiveness of this diagnosis.

Symptomatic treatment of these patients includes immunotherapies such as plasmapheresis, IVIG, rituximab, and steroids. These therapies are safe and patients are responsive with symptomatic relief though there is variability in treatment efficacy and outcomes.

## Conclusions

The neurological complications of anti-GAD antibodies are multiple and vary in presentation, progression, and responsiveness to treatments. Our two clinical cases exhibit the broad neurological presentations that a patient can possess. The variations in clinical presentation of anti-GAD antibodies make the discovery of these antibodies difficult. Additionally, these symptoms often mimic or present similarly to other common neurologic and autoimmune diseases. Thus, the discovery of these anti-GAD antibodies is crucial for treatment and control of symptoms. Clinicians must be vigilant when providing care to patients with complex clinical presentations. Early diagnosis is imperative to providing patients with adequate treatment to achieve symptom relief, even if at varying levels of improvement. This chronic disease course can greatly affect quality of life for patients. When patients are misdiagnosed with other neurological syndromes that may present like disorders associated with anti-GAD antibodies, they are further displaced from the possibility of symptomatic relief. Chronic suppression of these antibodies through immunosuppression is necessary for symptom improvement. In cases where patients present with complex neurological manifestations and workup is inconclusive, investigation with paraneoplastic panel may uncover the presence of anti-GAD antibodies and present new options for patient therapy.
